# Clinical presentations, complications, and outcomes of benoxinate topical ophthalmic anesthetic abuse: a retrospective cohort study

**DOI:** 10.1186/s12348-026-00636-4

**Published:** 2026-10-09

**Authors:** Suzan Ibrahim Sakr, Amira Ahmed Nayel, Dina Hazem ElHennawi, Fatma Ibrahim Saad, Alaa Atef Ghaith

**Affiliations:** 1 Cornea Clinic, Alexandria Ophthalmology Hospital, Ministry of Health and Population of Egypt, Alexandria, Egypt; 2 Clinical Pharmacy Department, Alexandria Ophthalmology Hospital, Ministry of Health and Population of Egypt, Alexandria, Egypt; 3 Ophthalmology Department, Faculty of Medicine, Alexandria University, Alexandria, Egypt

**Keywords:** Topical anesthetic abuse, Benoxinate toxicity, Ring infiltration, Corneal trauma, Anesthetics keratopathy

## Abstract

**Background:**

Topical anesthetic abuse is a concerning ophthalmic morbidity that may lead to irreversible visual loss. The primary goal of this study was to compare the clinical presentations, complications, healing duration and visual acuity changes between topical ophthalmic anesthetic users and non-users after superficial corneal trauma. The secondary goal was to correlate different variables related to the use of topical ophthalmic anesthetics with the final clinical outcomes.

**Methods:**

This retrospective cohort study reviewed files of patients with a positive history of superficial corneal trauma at Alexandria Ophthalmology Hospital Cornea Clinic, Alexandria, Egypt, between January 2021 and January 2026. Patients were divided according to the use of benoxinate eye drops into two groups (benoxinate users and benoxinate non-users). Data extracted included the patient’s job, the cause of the initial injury, the use of topical ophthalmic anesthetic (duration, frequency, and continuation after presentation), clinical picture, complications, healing duration, and visual outcomes.

**Results:**

A total of 181 eyes in 147 patients was included, 84 eyes in benoxinate users and 97 eyes in benoxinate non-users. Welders (76.2%), and males (89.3%) predominated in the benoxinate group (*p* < 0.001). The mean duration of benoxinate use was 11.67 (2–50) days. Clinical manifestations, particularly corneal melting (15 eyes, 17.9%), eyelid contact dermatitis (9 eyes, 10.7%), ring infiltration (26 eyes, 31.0%), complications as corneal vascularization (7 eyes, 8.3%), and perforation (17 eyes, 20.2%) were significantly higher in benoxinate users. Mean difference in visual acuity before and after treatment was significantly lower in benoxinate users (0.18 ± 0.25) than in benoxinate non-users (0.34 ± 0.23), (*p* < 0.001). Mean healing duration was markedly longer in benoxinate users (41.05 (4– 100)) days compared with the benoxinate non-users (7.93 (3–21)) days (*p* < 0.001). Adherent leukoma was observed only in patients who continue using benoxinate after starting treatment (15 eyes, 48.4%).

**Conclusions:**

Abusing topical ophthalmic anesthetics is associated with severe, permanent, and sight-threatening outcomes. Frequent and prolonged use of topical ophthalmic anesthetics aggravates ocular complications, and delays healing.

## Background

Cornea is one of the most densely innervated body tissues in the human body. Consequently, corneal abrasions, ultraviolet keratopathy and contact lens-related keratitis are often associated with significant pain [1]. Patients searching for pain relief can obtain ophthalmic anesthetics without a prescription worldwide [2, 3]. Furthermore, some ophthalmologists prescribe them after some corneal surgical procedures, e.g., photorefractive keratectomy [4].

Ophthalmic anesthetics are commonly used in daily ophthalmologic practice for diagnostic and therapeutic procedures. Topical ophthalmic anesthetics (TOA) are typically safe when used appropriately. However, their abuse could cause refractory corneal epithelial defects, stromal infiltration, severe vision-threatening adverse effects like degenerative keratopathy, corneal melting, and perforation [5]. On the cellular level, their abuse may result in inhibition of corneal cell growth, delay in cell proliferation, morphological abnormalities, and decreased viability of these cells, leading to severe ocular surface toxicity [6].

The clinical picture of ophthalmic anesthetics abuse may be misdiagnosed as microbial keratitis, e.g., ring infiltrates described in association with topical anesthetic abuse simulating that in acanthamoeba keratitis [7]. Moreover, the ocular or adnexal tissues toxicity manifested by cutaneous changes, conjunctivitis, and infectious complications are comparable to different allergic and immune-related lid disorders [8].

Although impacts of the ophthalmic anesthetics have been documented in literature, little evidence is available on the complications that may exist due to their abuse and their relation to the duration and frequency of their use [9].

This study described different clinical presentations, visual outcomes, healing duration and complications of topical anesthetic abuse after superficial corneal trauma at Alexandria Ophthalmology Hospital Cornea Clinic, Alexandria, Egypt. The duration, frequency, and continuation of using topical ophthalmic anesthesia drops were correlated with the final clinical outcomes.

## Methods

In this retrospective cohort study, the medical records of 147 patients (181 eyes) with a positive history of corneal trauma, including trauma with sharp objects, foreign bodies, contact lens, and ultraviolet exposure were reviewed. All patients were managed clinically by cornea specialists at Alexandria Ophthalmology Hospital Cornea Clinic, Alexandria, Egypt, between January 2021 and January 2026. Cases with herpetic corneal ulcers, corneal ulcers with negative history of corneal trauma, and files with incomplete data were excluded.

This study was conducted after approval from the Medical Research Ethics Committee, Ministry of Health and Population. The following data were collected for all patients: age, sex, job, the cause of initial injury, history of benoxinate topical anesthetic eye drops use, the duration (in days) of benoxinate eye drops use before first presentation, the continuation of benoxinate use even after starting treatment (yes: continue, no: did not continue), and frequency of drug use (high frequency: more than 6 times per day, low frequency: less than 6 times per day) [10].

Patients were divided according to their use of topical anesthetic eye drops (benoxinate hydrochloride 0.4%, Benox, EIPICO, Cairo, Egypt) eye drops into two groups (benoxinate users and benoxinate non-users).

**Fig. 1:**
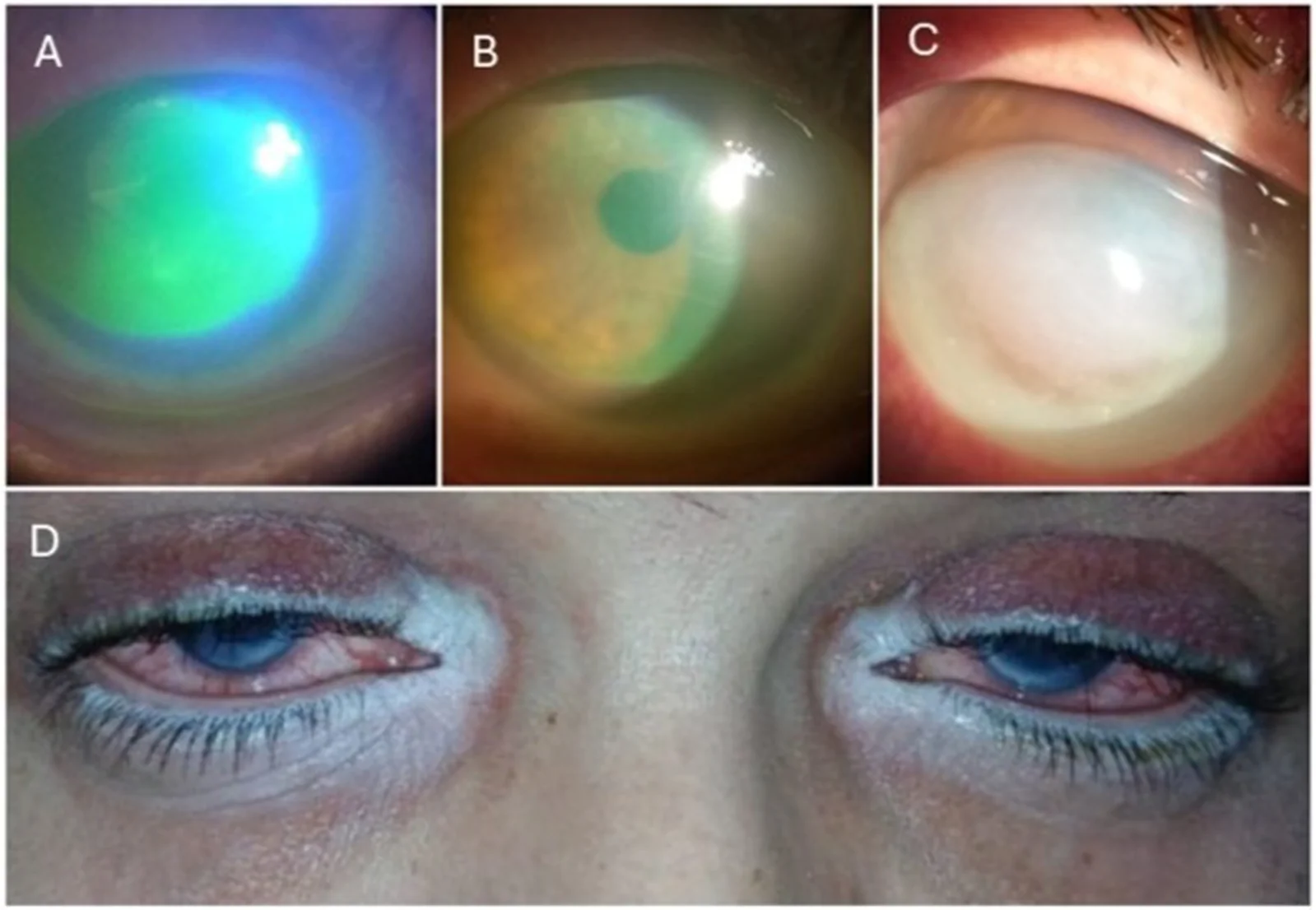
Clinical presentations of benoxinate toxicity (**A**) Fluorescein-stained corneal ulcer. (**B**) Corneal edema, demonstrating diffuse swelling and increased stromal thickness. (**C**) Corneal melting with progressive stromal dissolution and potential perforation. (**D**) Contact dermatitis with hyperemic irritated eyelids

**Fig. 2:**
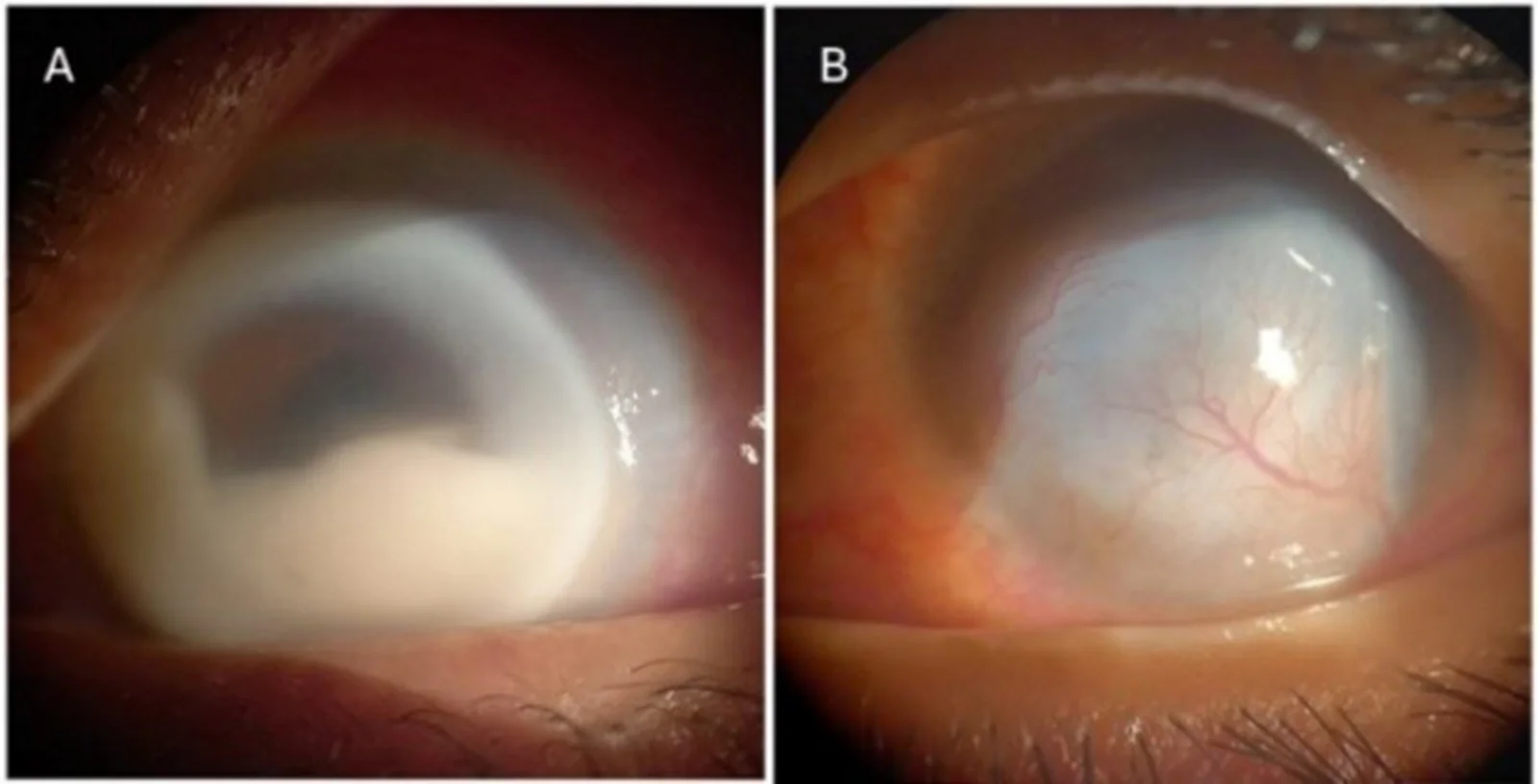
Complications of benoxinate toxicity (**A**) Corneal ulcer with secondary infection, hypopyon and ring infiltration. (**B**) Corneal neovascularization with blood vessel growth penetrating corneal stroma with stromal opacity

**Fig. 3:**
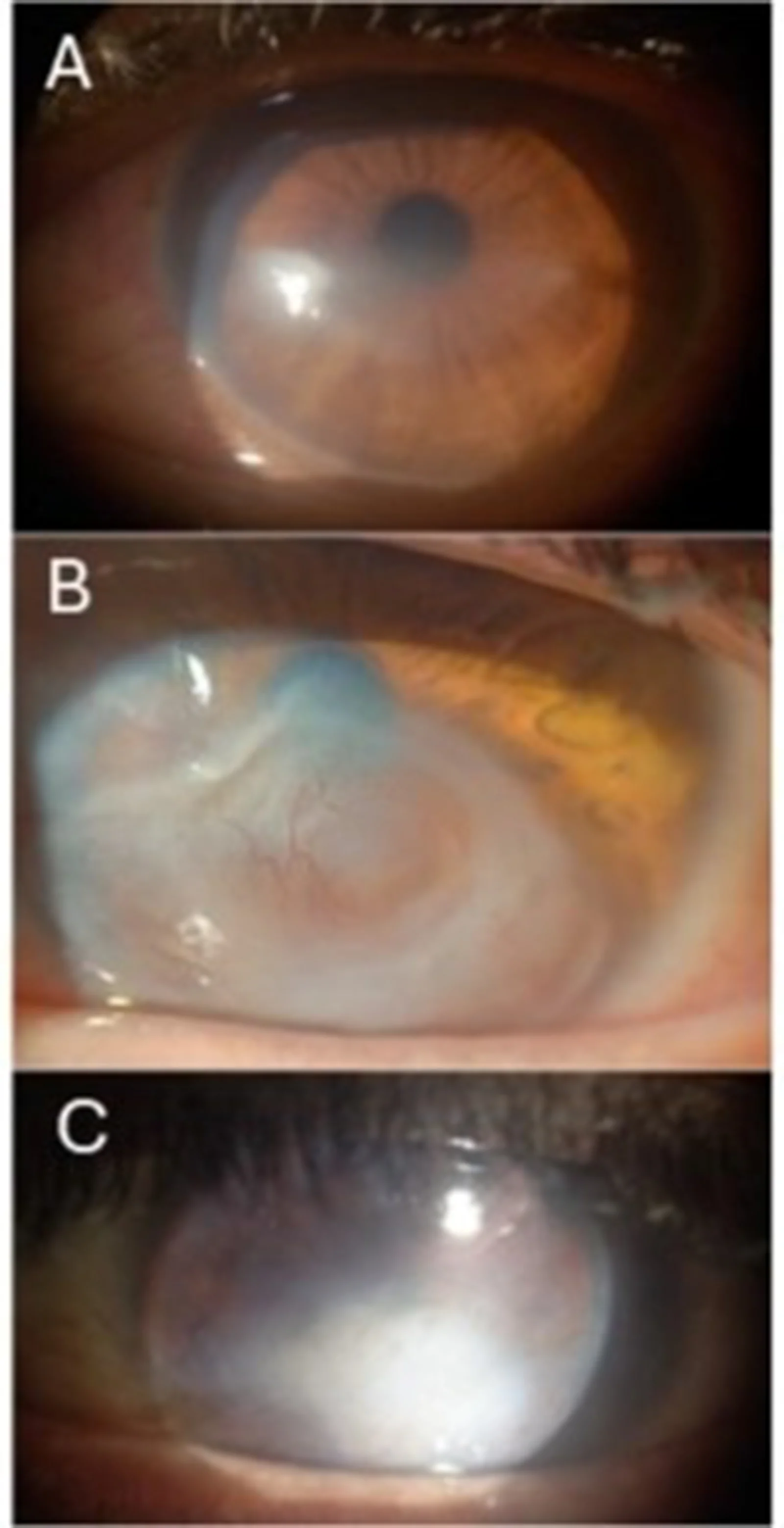
Final cornea clarity after benoxinate toxicity (**A**) Peripheral corneal opacity. (**B**) Central corneal opacity with neovascularization. (**C**) Adherent leukoma

Clinical presentation data studied included lid examination, presence of punctate epithelial erosions, corneal epithelial defect, corneal edema, hypopyon, characteristics of infiltration, keratic precipitates appearance and occurrence of corneal melting (Fig. 1). Clinical outcome data including complications (neovascularization, secondary infection or perforation) (Fig. 2), final cornea clarity (clear cornea, corneal opacity, and adherent leukoma) (Fig. 3), healing duration, and best-corrected visual acuity (BCVA) were collected for all patients.

The BCVA was measured as decimal values of Snellen fractions at presentation (BCVA at presentation) and at the last visit after treatment (post-treatment BCVA). The change in BCVA is the difference between post-treatment and BCVA at presentation. The BCVA decimal values smaller than 0.02, were described by counting fingers (CF), hand motion (HM), light perception (PL), and no light perception (NPL). The CF and HM were given the decimal value of 0.014 and 0.0052, respectively, according to the Freiburg Visual Acuity Test (FrACT) [11]. Moreover, the decimal values of 0.0016 and 0.0010 were assigned for the PL and NPL, respectively, which corresponds to their conversion decimals of logMAR values of 2.8 and 3.0, respectively [12].

**Fig. 4:**
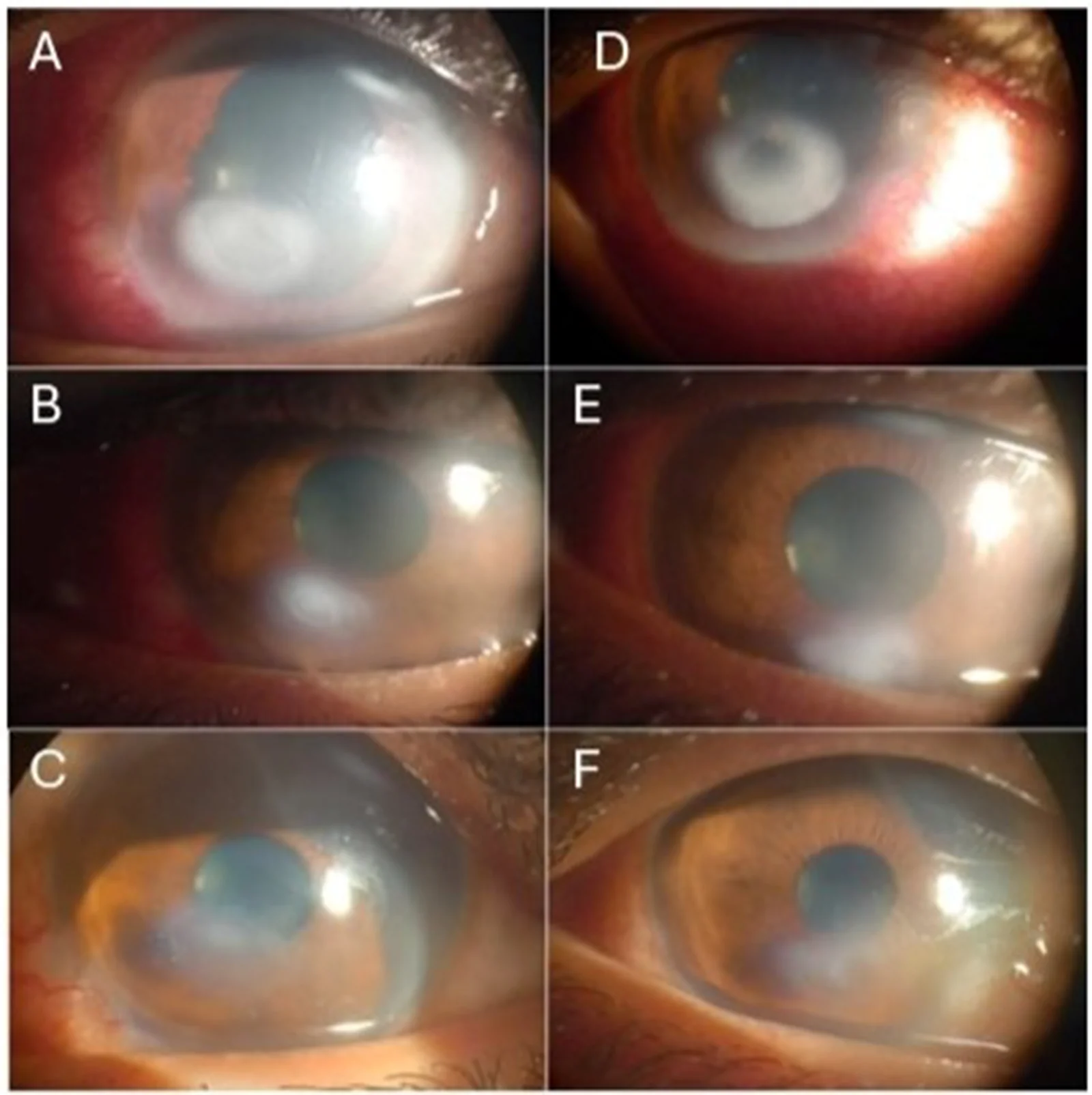
The healing process after benoxinate toxicity (**A-C**) Right eye healing process. (**D-E**) Left eye healing process

In this study, the management for all patients included immediate discontinuation of the topical anesthetic, broad spectrum topical antibiotic (Moxifloxacin), frequent preservative-free artificial tears together with systemic analgesics. Supportive treatment as systemic vitamin C and Doxycycline were prescribed in cases presented with corneal melting. Autologous serum and bandage contact lenses were prescribed in sterile resistant corneal ulcers. Eyelid contact dermatitis was managed with topical mixed antibiotics and steroids eye ointment. Moreover, all cases with secondary infection underwent corneal cultures. Empiric antibiotic therapy is initiated immediately after corneal scrapings are performed, awaiting culture and sensitivity results for guiding the antimicrobial therapy. The healing process after benoxinate toxicity was shown in Fig. 4. Cases with corneal melting and perforation were referred for emergency corneal grafting.

Data were fed to the computer and analyzed using the IBM SPSS software package version 27.0. (Armonk, NY: IBM Corp, released in 2020) . Categorical data were represented as numbers and percentages. **Chi-square test** was applied to compare the two groups. Alternatively, **Fisher’s Exact correction** test was applied when more than 20% of the cells had expected counts less than 5. Alternatively, the **Monte Carlo correction** test was applied when more than 20% of the cells had an expected count of less than 5. For continuous data, they were tested for normality by the **Shapiro-Wilk test**. Quantitative data were expressed as range (minimum and maximum), mean, standard deviation, median, and interquartile range (IQR). On the other hand, **the Mann-Whitney test** was used to compare two groups for not normally distributed quantitative variables. Significance of the obtained results was judged at the 5% level.

## Results

A total of 181 eyes in 147 patients were included in this study and were divided into two groups according to the use of topical benoxinate anesthetic: the benoxinate user group (*n* = 84) and the Benoxinate non-user group (*n* = 97). Table 1 shows that benoxinate use was significantly more common among males (89.3%) compared with benoxinate non-users (44.3%) (*p* < 0.001). The age of benoxinate users was also significantly older than the benoxinate non-users.

In terms of occupation, the majority of benoxinate users were welders (76.2%), while most patients in the benoxinate non-users were non-welders (85.6%) (*p* < 0.001). Predisposing factors differed significantly between the two groups; ultraviolet exposure and foreign body removal were more frequent among benoxinate user group, whereas sharp object injuries and contact lens-related injuries were more common in the benoxinate non-user group.

**Table 1:** Comparison between benoxinate user and benoxinate non-user groups according to demographic parameters

	Total (*n* = 181)	Benoxinate user (*n* = 84)	Benoxinate non-user (*n* = 97)	*p*
**Sex**				
Male	118 (65.2%)	75 (89.3%)	43 (44.3%)	> 0.001^*^
Female	63 (34.8%)	9 (10.7%)	54 (55.7%)	
**Age (years)**				
Min. – Max.	4.0–72.0	18.0–67.0	4.0–72.0	> 0.001^*^
Mean ± SD.	32.30 ± 15.54	39.15 ± 10.89	26.59 ± 16.55	
Median (IQR)	31.0 (22.0–41.0)	38.0 (32.0–45.0)	22.0 (16.0–32.0)	
**Occupation**				
Welder	78 (43.1%)	64 (76.2%)	14 (14.4%)	> 0.001^*^
Non- welder	103 (56.9%)	20 (23.8%)	83 (85.6%)	
**Laterality**				
Unilateral	113 (76.9%)	40 (64.5%)	73 (85.9%)	0.002^*^
Bilateral	34 (23.1%)	22 (35.5%)	12 (14.1%)	
**Predisposing factor**				
Sharp object	48 (26.5%)	4 (4.8%)	44 (45.4%)	< 0.001^*^
Ultraviolet exposure	36 (19.9%)	36 (42.9%)	0 (0.0%)	< 0.001^*^
Foreign body removal	42 (23.2%)	35 (41.7%)	7 (7.2%)	< 0.001^*^
Contact lens	47 (26.0%)	8 (9.5%)	39 (40.2%)	< 0.001^*^
Chemical burn	8 (4.4%)	1 (1.2%)	7 (7.2%)	^FE^*p*=0.070

IQR: Inter quartile range SD: Standard deviation U: Mann Whitney test

χ^2^: Chi square test FE: Fisher Exact test MC: Monte Carlo test

p: p value for comparing between the studied groups

*: Statistically significant at *p* ≤ 0.05

Certain clinical manifestations were significantly more frequent in the benoxinate user group than in non-users. Particularly, benoxinate users had 20 times higher odds of corneal melting (*p* < 0.001, OR = 20.87, 95% CI: 2.693-161.747), 6 times higher odds of presenting with hypopyon (*p* = 0.002, OR = 6.267, 95% C.I: 1.734–22.647), and nearly 11 times higher odds of developing eyelid contact dermatitis (*p* = 0.006, OR = 11.520, 95% CI:1.428–92.947).

Regarding the pattern of corneal infiltration, significant differences were observed in the pattern of corneal infiltration between the two groups, with ring infiltration occurring exclusively in the benoxinate user group (*p* < 0.001). Severe complications such as corneal perforation and corneal vascularization were significantly more common in the benoxinate user group (*p* < 0.001), and (*p* = 0.004), respectively.

**Fig. 5:**
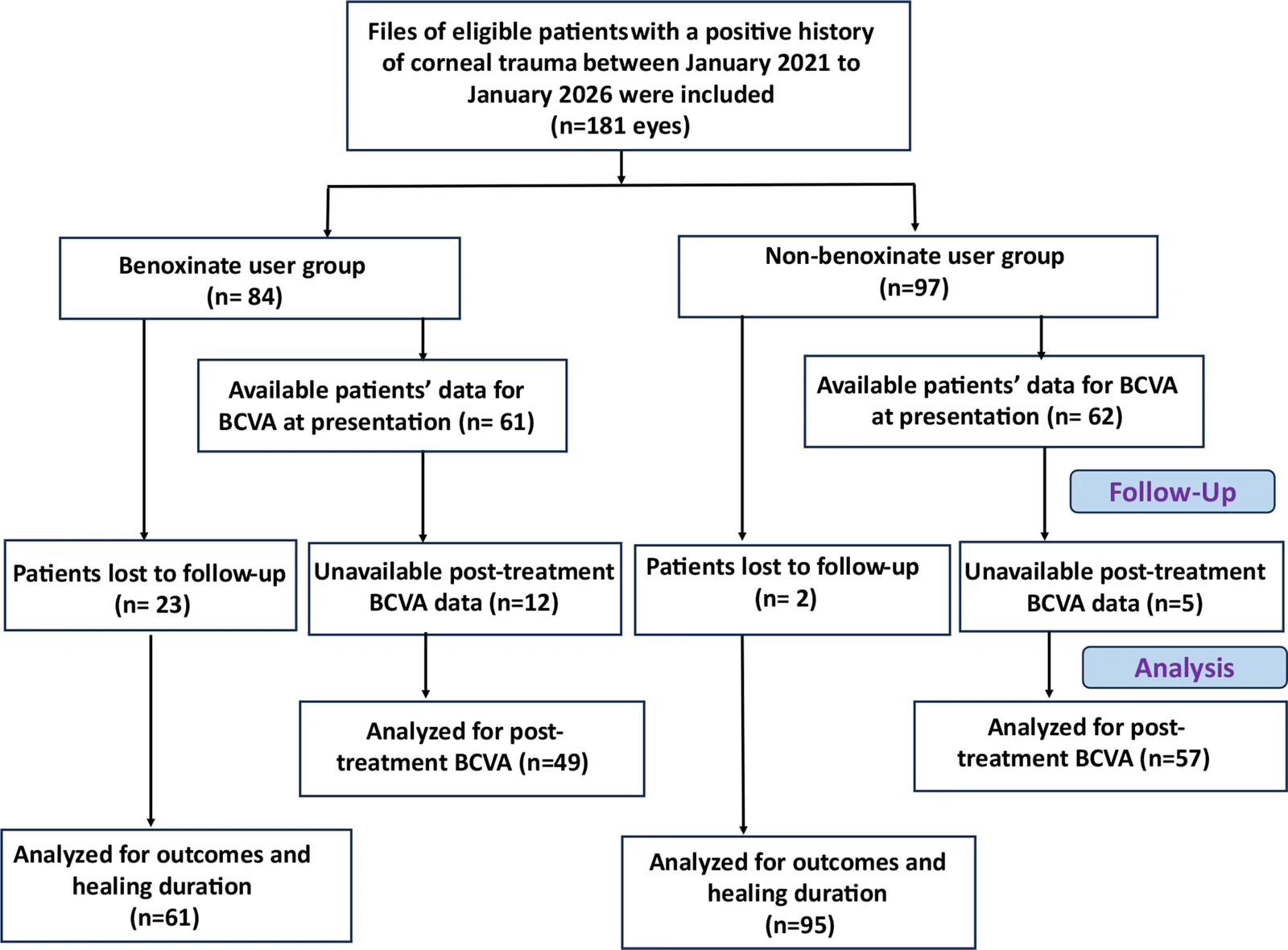
A flow-chart diagram of the study profile

A flow- chart diagram of the study profile is presented in Fig. 5. Twenty-three cases were lost to follow-up in the benoxinate use group and 2 cases in the benoxinate non-user group. Adherent leukoma occurred solely in the benoxinate use group (*p* < 0.001). Healing duration was markedly longer in benoxinate users (mean 41.05 ± 31.72 days) compared with benoxinate non-users (mean 7.93 ± 4.87 days) (*p* < 0.001), demonstrating a large effect size (correlation coefficient *r* = 0.645; 95% C.I: 0.543–0.728). (Table 2)

**Table 2:** Comparison between benoxinate user and benoxinate non-user groups according to clinical manifestations, complications, healing duration, and final outcomes

	Total (*n* = 181)	Benoxinate user (*n* = 84)	Benoxinate non-user (*n* = 97)	*p*	Univariate OR (LL – UL 95%CI)
**Clinical presentation**					
Punctate epithelial erosion	24 (13.3%)	10 (11.9%)	14 (14.4%)	0.617	0.801 (0.336–1.912)
Corneal ulcer	156 (86.2%)	73 (86.9%)	83 (85.6%)	0.795	1.119 (0.478–2.619)
Corneal edema	55 (30.4%)	31 (36.9%)	24 (24.7%)	0.076	1.779 (0.938–3.373
Corneal melting	16 (8.8%)	15 (17.9%)	1 (1.0%)	> 0.001^*^	20.870 (2.693–161.747)
KPs	2 (1.1%)	2 (2.4%)	0 (0.0%)	^FE^*p*=0.214	NA
Hypopyon	17 (9.4%)	14 (16.7%)	3 (3.1%)	0.002^*^	6.267 (1.734–22.647)
Eyelid contact dermatitis	10 (5.5%)	9 (10.7%)	1 (1.0%)	^FE^*p*=0.006^*^	11.520 (1.428–92.947)
**Infiltration**					
Absent	111 (61.3%)	43 (51.2%)	68 (70.1%)	0.009^*^	2.236 (1.215–4.114)
Present	**70 (38.7%)**	**41 (48.8%)**	**29 (29.9)**		
Peripheral	15 (8.3%)	6 (7.1%)	9 (9.3%)	0.603	0.752 (0.16–2.208)
Central	22 (12.2%)	8 (9.5%)	14 (14.4%)	0.313	0.624 (0.248–1.570)
Perineural	6 (3.3%)	0 (0.0%)	6 (6.2%)	^FE^*p*=0.031^*^	NA
Ring	26 (14.4%)	26 (31.0%)	0 (0.0%)	< 0.001^*^	NA
Ring & central	1 (0.6%)	1 (1.2%)	0 (0.0%)	^FE^*p*=0.464	NA
**Complications**					
None	120 (66.3%)	50 (59.5%)	70 (72.2%)	0.073	0.567 (0.305–1.057)
Secondary infection	57 (31.5%)	30 (35.7%)	27 (27.8%)	0.255	1.440 (0.767–2.703)
Vascularization	7 (3.9%)	7 (8.3%)	0 (0.0%)	^FE^*p*=0.004^*^	NA
Corneal perforation	17 (9.4%)	17 (20.2%)	0 (0.0%)	> 0.001^*^	NA
**Final Outcome**	(***n*** **= 156)**	(***n*** **= 61)**	(***n*** **= 95)**		
Clear cornea	89 (57.0%)	22 (36.1%)	67 (70.5%)	< 0.001^*^	0.236 (0.119–0.467)
Peripheral corneal opacity	22 (14.1%)	8 (13.1%)	14 (14.7%)	0.776	0.873 (0.343–2.225)
Central corneal opacity	30 (19.2%)	16 (26.2%)	14 (14.7%)	0.076	2.057 (0.920–4.599)
Adherent leukoma	15 (9.6%)	15 (24.6%)	0 (0.0%)	< 0.001^*^	NA
**Healing duration (days)**	(***n*** **= 156)**	(***n*** **= 61)**	(***n*** **= 95)**		
Min. – Max.	3.0–100.0	4.0–100.0	3.0–21.0	> 0.001^*^	*r* = 0.645 (0.543–0.728)
Mean ± SD.	20.88 ± 25.82	41.05 ± 31.72	7.93 ± 4.87		
Median (IQR)	10.0 (5.0–21.0)	30.0 (14.0–60.0)	7.0 (4.0–10.0)		

IQR: Inter quartile range, SD: Standard deviation, χ^2^: Chi square test, FE: Fisher Exact test, MC: Monte Carlo test, OR: Odds Ratio, r: Correlation Coefficient (Mann Whitney test), LL: Lower limit, UL: Upper limit, p: p value for comparing between the studied groups, *: Statistically significant at *p* ≤ 0.05, NA: Non-applicable

Regarding visual acuity as shown in Table 3, the results indicate that the use of benoxinate was associated with significantly worse best corrected visual acuity (BCVA) both before and after treatment compared with non-using benoxinate (*p* < 0.001). Although BCVA improved after treatment in both groups, the degree of improvement was significantly lower in the benoxinate user group (*p* < 0.001, *r* = 0.309; CI: 0.159 − 0.444). Visual acuity measurements were unavailable for 33 eyes in pediatric patients.

**Table 3:** Comparison between Benoxinate user and Benoxinate non-user groups according to Vision

Visual Acuity (Decimal)	Total	Benoxinate user	Benoxinate non-user	*p*	*r* (LL – UL 95% CI)
**BCVA at presentation**	(*n* = 123)	(*n* = 61)	(*n* = 62)		
Min. – Max.	0.0–0.80	0.01–0.50	0.00–0.80	> 0.001^*^	
Mean ± SD.	0.15 ± 0.19	0.08 ± 0.13	0.21 ± 0.22		0.285 (0.134 − 0.423)
Median (IQR)	0.05 (0.01–0.30)	0.01 (0.01–0.10)	0.10 (0.01–0.40)		
**Post-treatment BCVA**	(***n*** **= 106)**	(***n*** **= 49)**	(***n*** **= 57)**		
Min. – Max.	0.0–1.0	0.00–0.90	0.00–1.0	> 0.001^*^	
Mean ± SD.	0.42 ± 0.36	0.25 ± 0.32	0.56 ± 0.33		0.369 (0.224 − 0.497)
Median (IQR)	0.40 (0.05–0.80)	0.05 (0.01–0.40)	0.60 (0.20–0.80)		
**BCVA change**					
Min. – Max.	-0.50–0.95	-0.50–0.70	0.00–0.95	< 0.001^*^	
Mean ± SD.	0.27 ± 0.25	0.18 ± 0.25	0.34 ± 0.23		0.309 (0.159 − 0.444)
Median (IQR)	0.22 (0.04–0.43)	0.05 (0.00–0.37)	0.30 (0.19–0.50)		

IQR: Inter quartile range, r: Correlation Coefficient (Mann Whitney test), LL: Lower limit, UL: Upper limit, SD: Standard deviation, U: Mann Whitney test, BCVA: best-corrected visual acuity, p: p value for comparing between the studied groups, *: Statistically significant at *p* ≤ 0.05

**Fig. 6:**
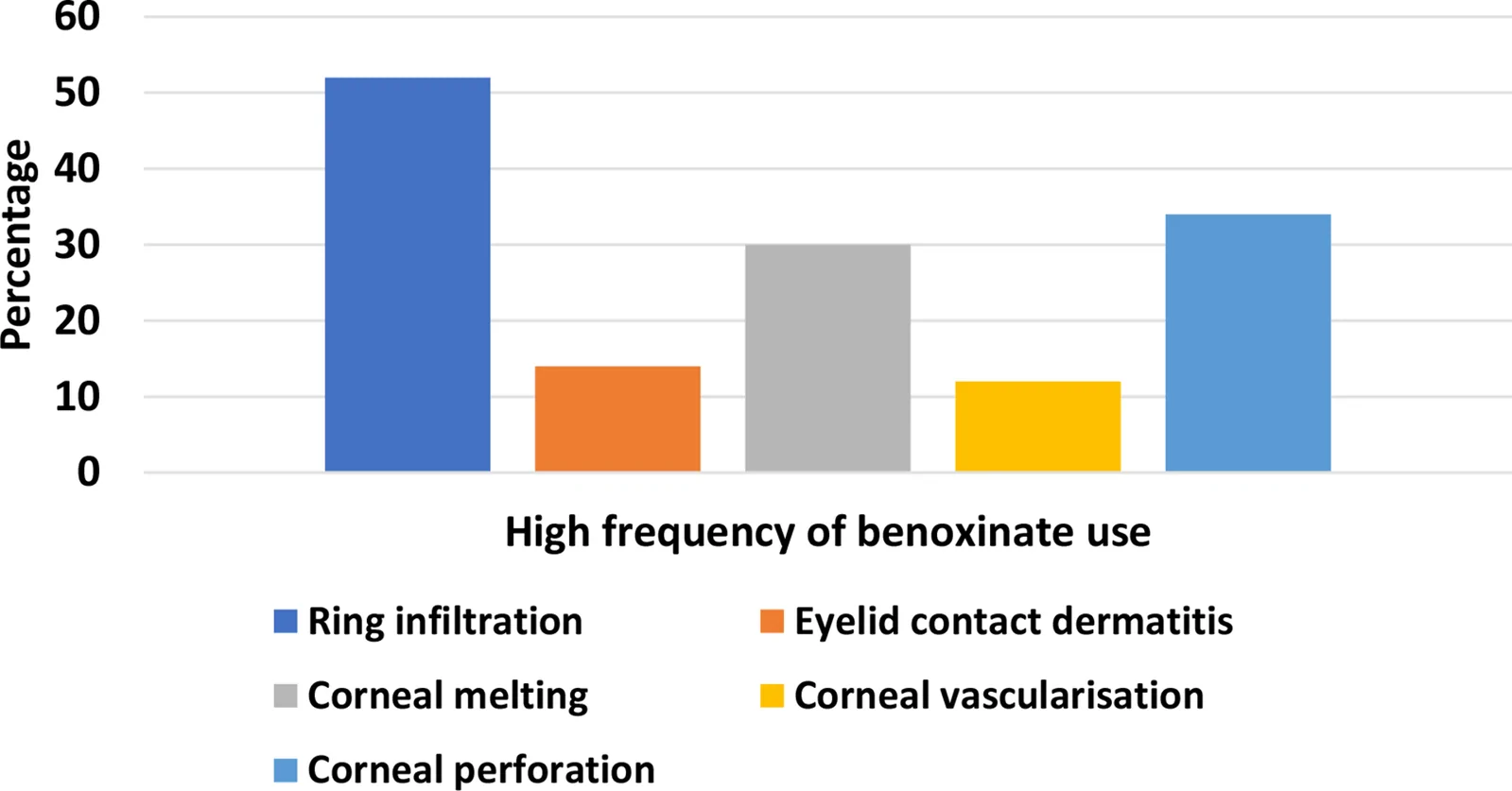
The effect of high frequency of benoxinate use on clinical presentation and complications

Table 4 demonstrates that 50 eyes (59.5%) out of a total of 84 eyes had a high frequency of benoxinate use. Patients with high frequency of benoxinate use showed a significant increase in certain clinical presentations; corneal melting (15 eyes, 30.0%, *p* < 0.001), ring infiltration (26 eyes, 52.0%, *p* < 0.001), and demonstrated more than 2 times higher odds of eyelid contact dermatitis (7 eyes, 14.0%, *p* = 0.25, OR = 2.60; CI: 0.507–13.384). Corneal perforation (17, eyes, 34.0%) occurred only in high frequency users (*p* < 0.001) (Fig. 6). High frequency benoxinate users exhibited more than 4 folds higher odds of developing vascularization (*p* = 0.173, OR = 4.500; CI: 0.507–39.199) than their low frequency users’ counterparts.

**Table 4:** Comparison between Benoxinate use (high and low frequency) regarding clinical presentations, and complications

	Frequency of Benoxinate use		*p*	Univariate OR (LL – UL 95%C.I)
	Low (*n* = 34)	High (*n* = 50)		
**Clinical presentations**				
Corneal melting	**0 (0.0%)**	15 (30.0%)	< 0.001^*^	NA
Eyelid contact dermatitis	2 (5.9%)	7 (14.0%)	0.252	2.605 (0.507–13.384)
Ring Infiltration	**0 (0.0%)**	26 (52.0%)	< 0.001^*^	NA
**Complications**				
Vascularization	1 (2.9%)	6 (12.0%)	0.173	4.500 (0.517–39.199)
Corneal perforation	**0 (0.0%)**	17 (34.0%)	< 0.001^*^	NA

OR: Odds Ratio, LL: Lower limit, UL: Upper limit, p: p value for comparing between the studied groups, *: Statistically significant at *p* ≤ 0.05, NA: Non-applicable

Figure 6 demonstrates that high frequency benoxinate use was associated with a high occurrence of severe clinical manifestations and complications, including ring infiltration, corneal melting, and corneal perforation. These findings suggest that repeated topical anesthetic exposure may exacerbate corneal toxicity by impairing epithelial healing, reducing corneal sensation, and promoting stromal damage. The predominance of severe complications among frequent users supports the importance of limiting unsupervised topical anesthetic use.

**Fig. 7:**
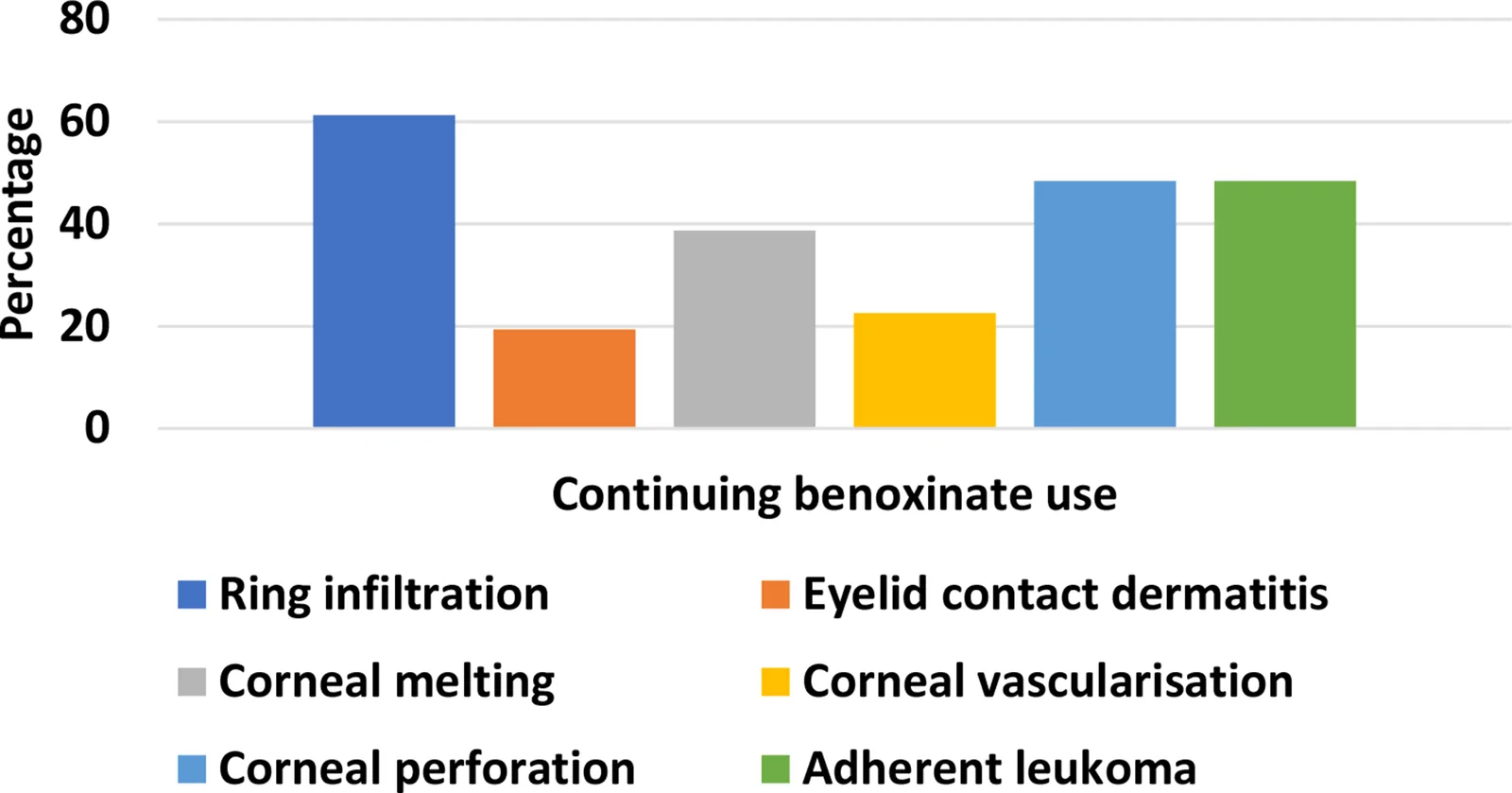
The effect of continuing benoxinate use on clinical presentation, complications, and final outcomes

Sixty-one eyes continued follow-up and approximately half of them (31, 50.8%) continued using the anesthetic drops after starting treatment (Table 5). Patients who continue to use benoxinate showed a significant increase in certain clinical manifestations like eyelid contact dermatitis (6 eyes, 19.4%), and ring infiltration (19 eyes, 61.3%). Corneal melting (12 eyes, 38.7%) vascularization (7 eyes, 22.6%), perforation (15 eyes, 48.4%) and adherent leukoma (15 eyes, 48.4%) occurred exclusively in those who continued using benoxinate eye drops (Fig. 7).

**Table 5:** Comparison between Benoxinate use (continuation and non-continuation) regarding clinical presentations, complications, and final outcomes

	Continue benoxinate use		*P*	Univariate OR (LL – UL 95%C.I)
	No (*n* = 30)	Yes (*n* = 31)		
**Clinical presentations**				
Corneal melting	**0 (0.0%)**	12 (38.7%)	< 0.001^*^	NA
Eyelid contact dermatitis	2 (6.7%)	6 (19.4%)	0.160	3.360 (0.621–18.186)
Ring Infiltration	2 (6.7%)	19 (61.3%)	< 0.001^*^	22.167 (4.447–110.487)
**Complications**				
Vascularization	**0 (0.0%)**	7 (22.6%)	0.011^*^	NA
Corneal perforation	**0 (0.0%)**	15 (48.4%)	< 0.001^*^	NA
**Final outcome**				
Adherent leukoma	**0 (0.0%)**	15 (48.4%)	< 0.001^*^	NA

OR: Odds Ratio, LL: Lower limit, UL: Upper limit, p: p value for comparing between the studied groups, *: Statistically significant at *p* ≤ 0.05, NA: Non-applicable

Figure 7 further highlights that continued benoxinate use after the onset of symptoms was associated with persistent disease progression and poorer clinical outcomes. Patients who continued using benoxinate experienced higher rates of corneal melting, perforation, and permanent corneal scarring, emphasizing that prompt discontinuation of the offending agent is a critical step in preventing irreversible ocular damage.

**Fig. 8:**
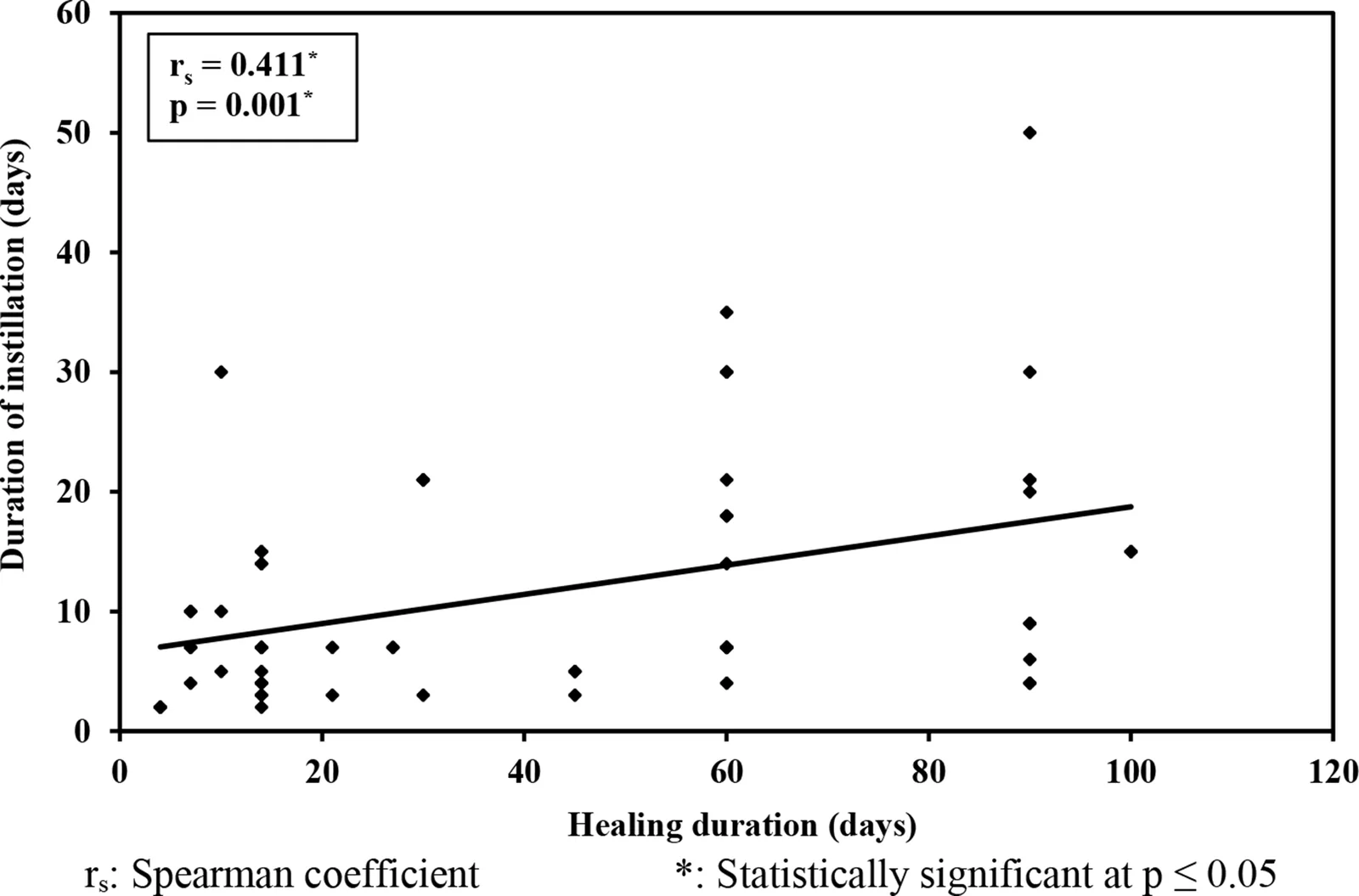
Correlation between duration of benoxinate use (days) and healing duration (days) in benoxinate group

**Fig. 9:**
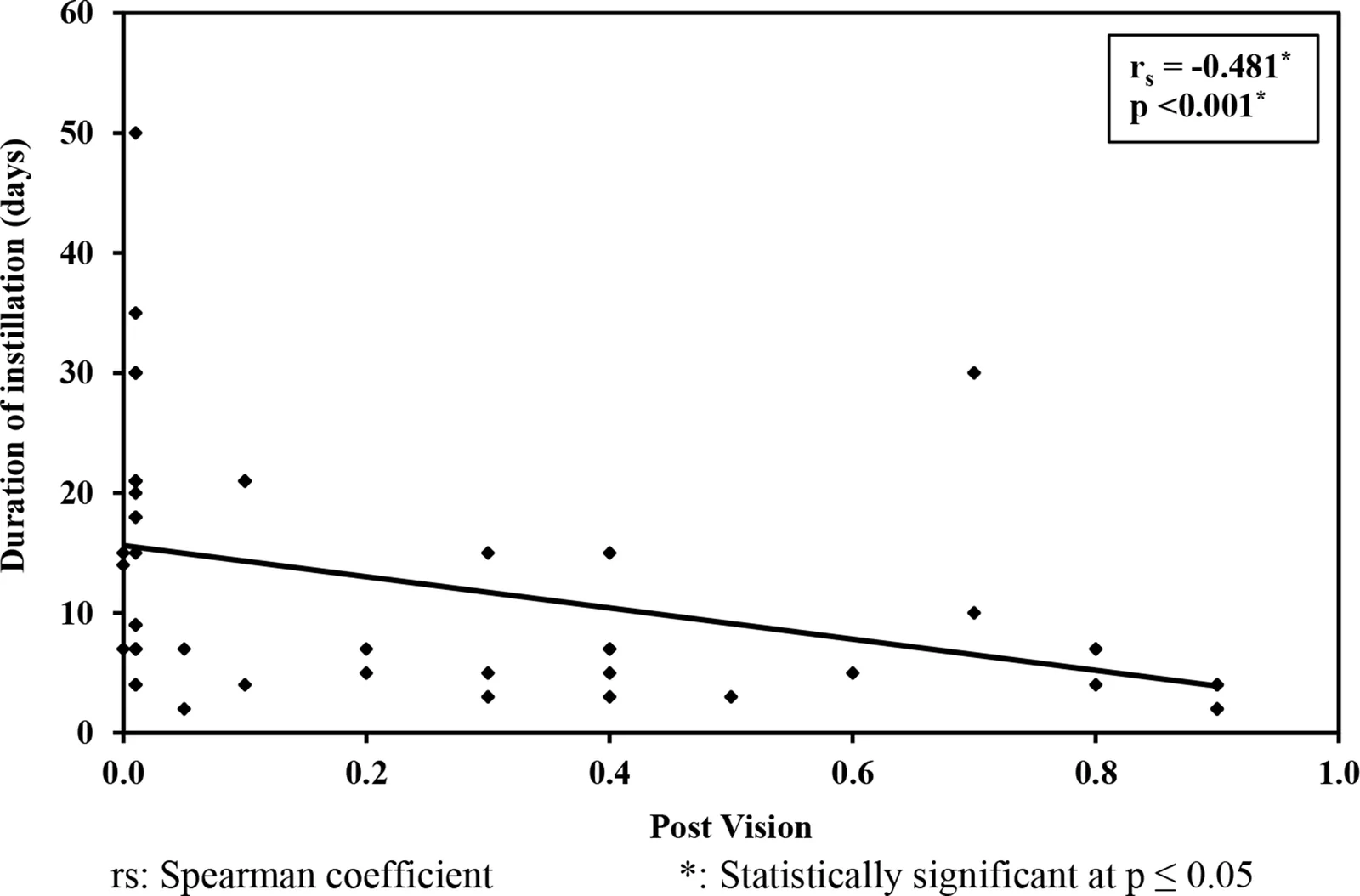
Correlation between duration of benoxinate use (days) and post-treatment BCVA in benoxinate group

**Fig. 10:**
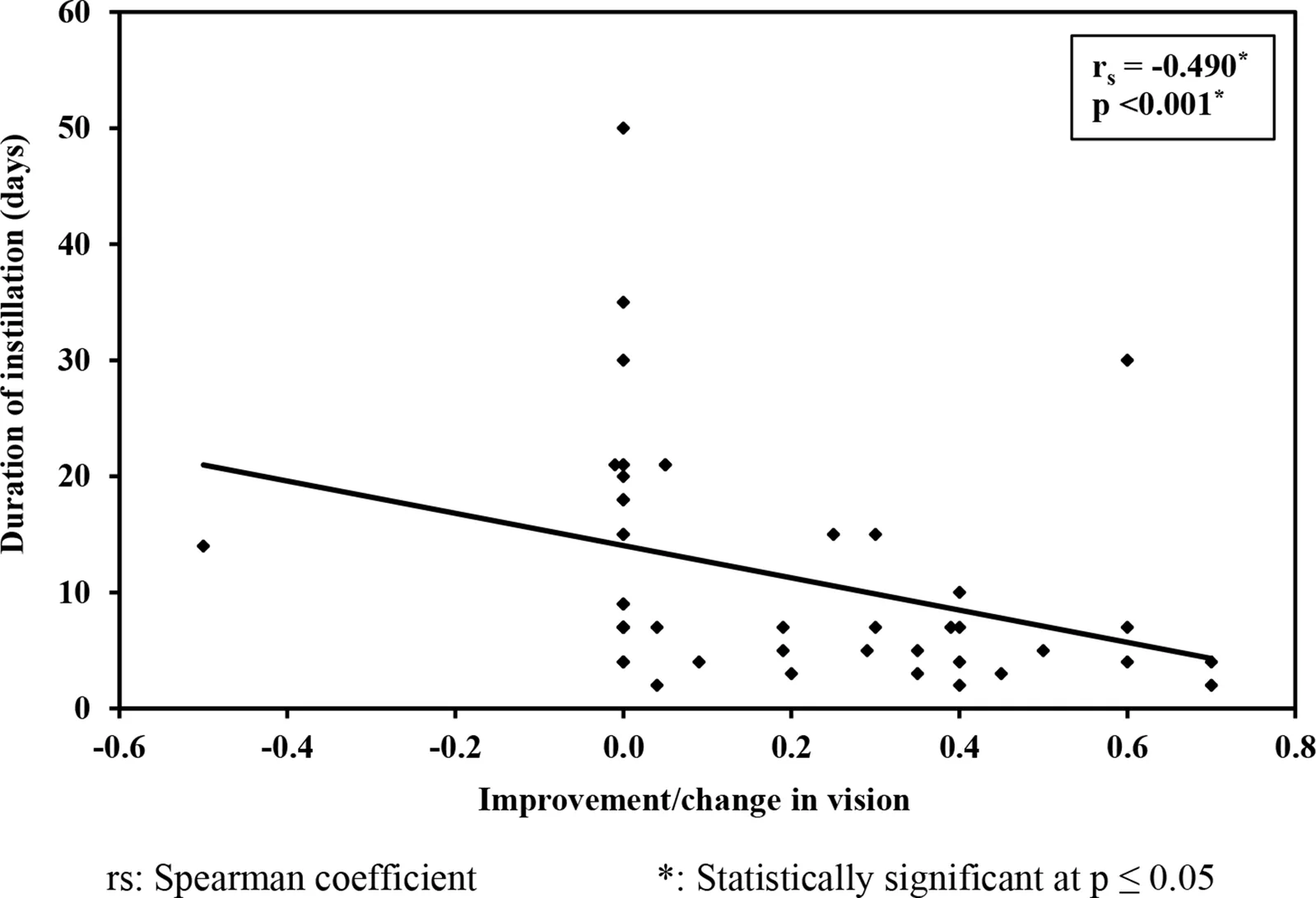
Correlation between duration of benoxinate use (days) and change in BCVA in benoxinate group

The duration of benoxinate use ranged between 2 and 50 days, with a mean duration of 11.67 ± 9.56 days. The correlation analysis demonstrated that increasing duration of benoxinate use was associated with prolonged healing time and poorer visual outcomes. Specifically, longer benoxinate exposure correlated positively with healing duration (*r* = 0.411, *p* = 0.001) (Fig. 8) and negatively with both post-treatment BCVA (*r* = − 0.481, *p* < 0.001) and improvement in BCVA (*r* = − 0.490, *p* < 0.001) (Figs. 9 and 10, respectively). These findings support the concept that prolonged topical anesthetic use contributes to cumulative corneal toxicity, delaying epithelial recovery and resulting in less favorable visual outcomes. This highlights the importance of early recognition and prompt discontinuation of benoxinate to minimize irreversible corneal damage.

**Fig. 11:**
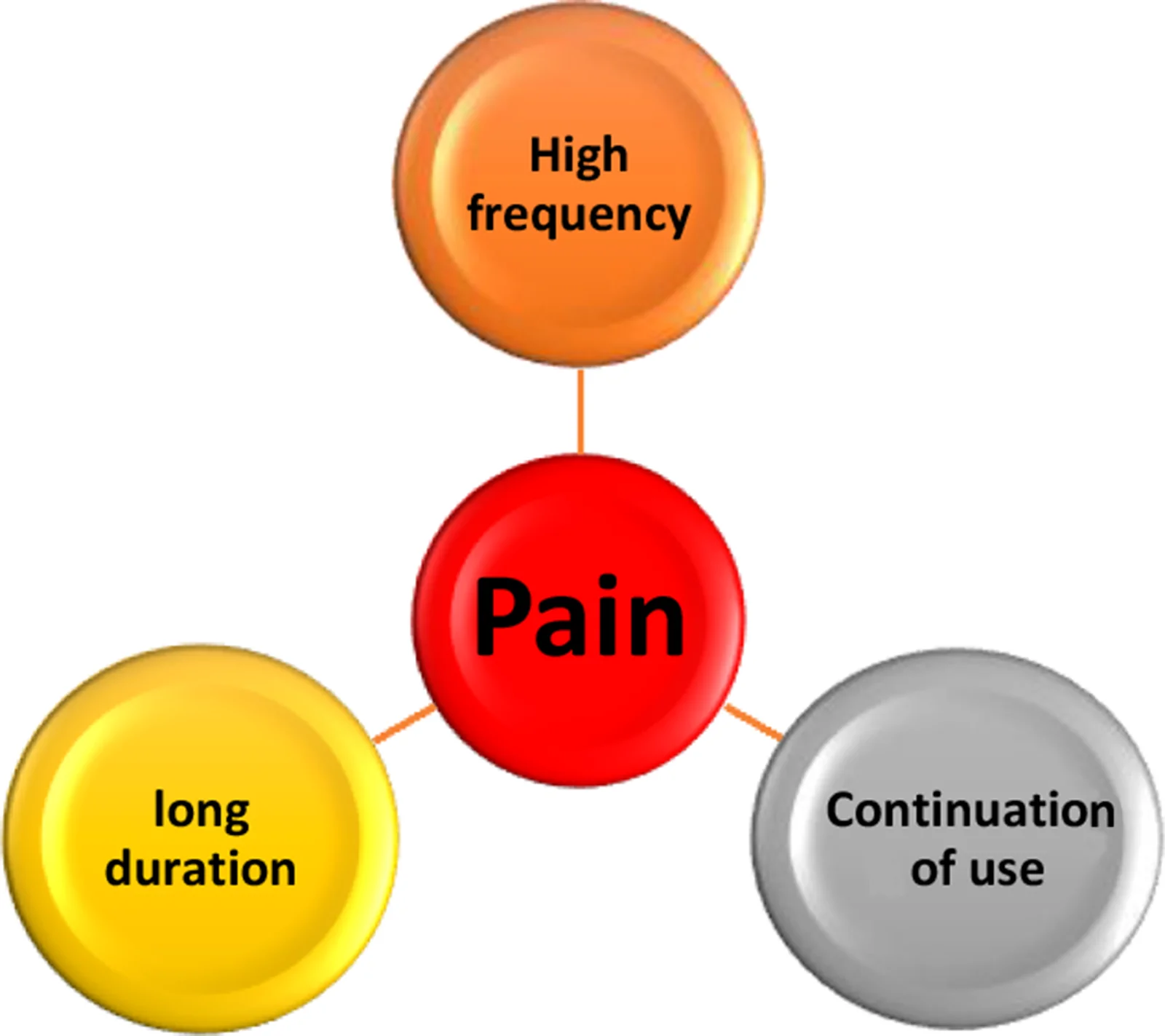
Topical benoxinate toxicity risk factors

Figure 11 summarizes the three principal factors contributing to the development and progression of topical benoxinate toxicity: high frequency of use, the prolonged duration of use before patients’ first presentation, and the continuation of benoxinate use even after starting treatment. As the ocular pain persists, these factors create a vicious cycle that accelerates corneal toxicity and increases the likelihood of vision-threatening complications.

## Discussion

Abuse of ophthalmic anesthetics is associated with severe ocular complications leading to permanent morbidities. Ocular anesthetic abuse is particularly common among male welders of working age. In this study, males are more affected than females, this is attributed to their working nature as welders. This study found that the percentage of welders were significantly higher in benoxinate uses (76.2%) over benoxinate non-uses (14.4%). Similarly, Sharifi et al. and Attar et al. showed that most affected individuals were welders [3, 13].

The current study recognized that the most common initial injuries leading to the abuse of topical anesthetics were exposure to ultraviolet rays (photokeratitis) and ocular surface foreign bodies. Similarly, Yagci et al., and Sharifi et al. reported the same initial injuries in their studies. Other causes were due to contact lens wear, dry eye disease, Bell’s palsy, chemical burns, corneal abrasion, and thermal burns [3, 5, 14].

Topical anesthetics have both dose- and time-dependent cytotoxic effects on corneal epithelial cells [15]. In this study, the mean duration of benoxinate use at the time of presentation was 11.67 days. Sharifi et al., reported the mean duration of tetracaine abuse was 9.03 days [8]. Erdem et al. and Tok et al. reported a mean duration of anesthetic abuse of 15 days and 18 days, respectively [16, 17]. This prolonged use of medication causes a significant delay in diagnosis and management.

In many patients, abuse of topical anesthetics may continue even after initiating treatment. The current study revealed the continuation of benoxinate use in 31 (50.8%) patients despite the recommendations to discontinue their use. Yagci et al. reported a series of 19 cases, 12 of whom had continued abuse of drops during their hospitalization time [5].

In the current study, the most common clinical findings were PEE, corneal epithelial defect, corneal edema, and ring corneal infiltrations. Similarly, Rosenwasser et al. reported six individuals with topical anesthetic toxicity suffering from PEDs and corneal stromal ring infiltrates. Of the six patients, five cases were mistakenly diagnosed and treated as Acanthamoeba keratitis due to ring infiltration that mimics acanthamoeba keratitis [18].

In this study, post-treatment visual acuity improved significantly compared with pre-treatment visual acuity. This finding is in agreement with previous studies reporting favorable visual outcomes following appropriate management of topical anesthetic abuse [13, 17].

Corneal anesthesia and toxicity may predispose to multiple complications as it compromises tear function by decreasing aqueous tear secretion, disturbing tear film stability, and decreasing blinking rate [18]. Multiple studies reported complications like secondary infection, corneal neovascularization, melting, and perforation, especially in cases with poor compliance [8, 13, 19]. In the current study, similar complications were significantly higher among non-compliant benoxinate uses, highlighting the need for regular follow-up and close monitoring.

Patients seek pain relief and often attempt to obtain ophthalmic anesthetics by any means. Pain control is the cornerstone of preventing progression of toxicity. Hence, it is strongly recommended to offer strong systemic analgesics together with topical cycloplegic agents to relax the ciliary muscles and the iris sphincter to stop the occurrence of a vicious cycle that, in turn, leads to exacerbation of serious complications.

**Fig. 12:**
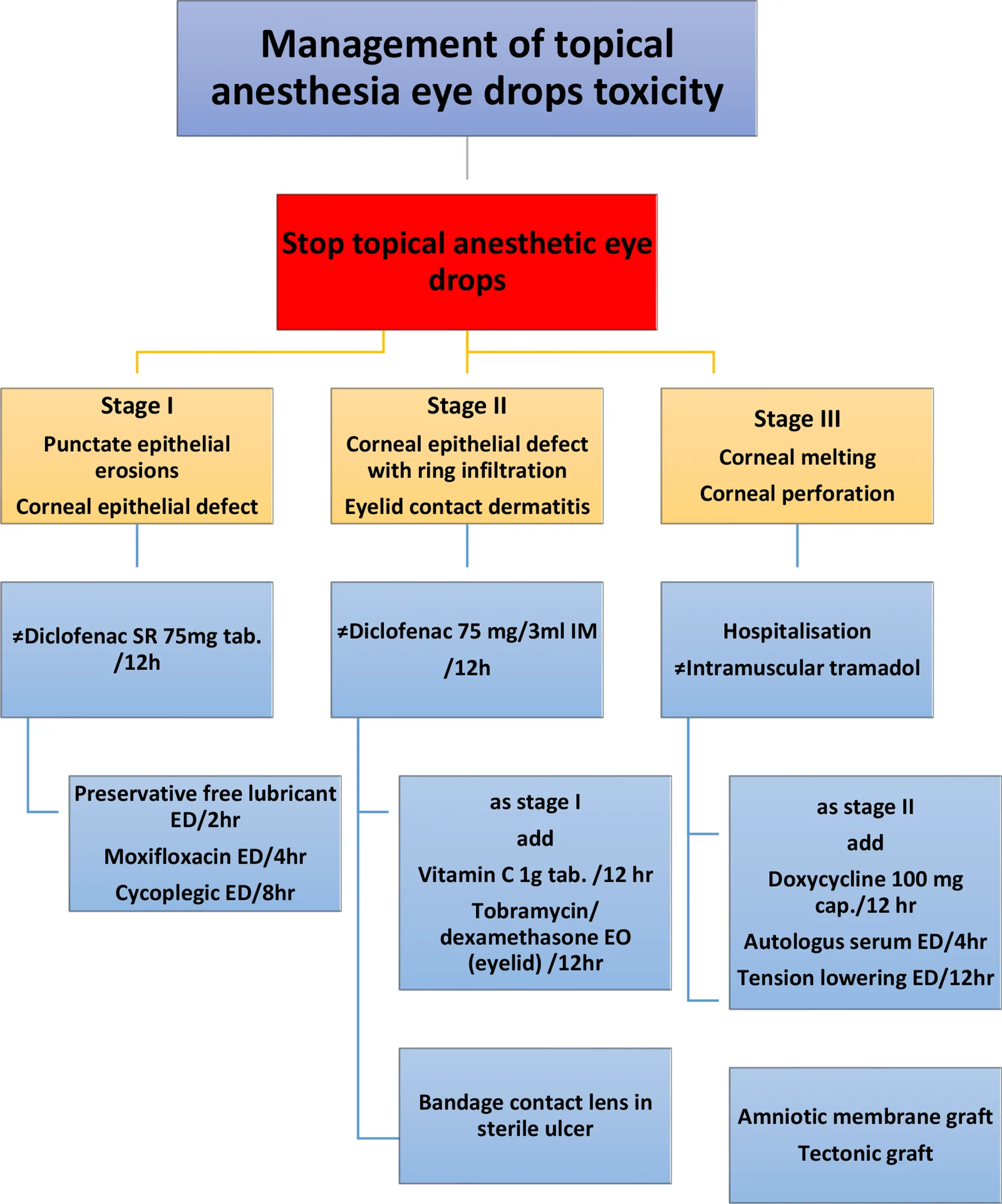
A stepwise algorithm for topical benoxinate toxicity management. ≠ Clinicians should be cautious about prescribing strong systemic analgesics, especially in case of elderly patients, patients suffering from renal or hepatic impairment, and patients with ischemic heart disease. The administration of strong analgesics should be under close clinical supervision

The strengths of this study include the comparative group that highlights the difference between complications, visual and final outcomes among benoxinate users and benoxinate non-users in corneal trauma. Furthermore, a proposed management algorithm for topical benoxinate toxicity was presented in Fig. 12. The algorithm integrates the findings of the present study with current evidence and expert recommendations to provide a practical, stepwise approach for clinicians. It emphasizes early recognition of topical anesthetic abuse, comprehensive assessment of corneal involvement and immediate discontinuation of benoxinate. Initiation of appropriate medical therapy was done according to the clinical staging at presentation including pain relief measurements as a keystone in benoxinate toxicity management. Surgical intervention was indicated in complicated cases like corneal melting and corneal perforation. The algorithm offers a structured framework that may facilitate earlier diagnosis, standardize management, and potentially improve visual outcomes in patients with topical anesthetic toxicity [8, 19–22].

The limitations of this study encompassed the marked difference in the loss to follow-up between the two groups (27% in the benoxinate user group versus 2% in the non-user group). Reasons for poor treatment adherence in benoxinate users may be attributed to the underestimation of benoxinate users to the risk of topical anesthetic eye drops use, the severity of their medical condition, or due to socioeconomic barriers. This differential attrition represents a potential source of attrition bias, as patients who were lost to follow-up may have had clinical outcomes that differed from those who completed the study. Consequently, the observed differences between the groups may have been overestimated or underestimated.

The sequelae of TOA toxicity are not definitely resolved by the emergency keratoplasty; rather, they extend beyond surgery and may persist as postoperative complications, including delayed epithelial healing, secondary glaucoma, and high incidence of graft rejection. Consequently, we advocate for systematic evaluation of the surgical outcomes in these cases as these issues warrant further investigation.

## Conclusions

In conclusion, abusing benoxinate topical ophthalmic anesthetics is associated with severe, permanent, sight-threatening and debilitating effects, including corneal melting, corneal opacity, and reduced visual acuity. The healthcare system should restrict the OTC access to TOA, especially among welders. It is also necessary to increase awareness of general ophthalmologists about early diagnosis and proper management of TOA toxicity. High frequency, prolonged duration of use and continuation of using TOA despite starting treatment are the three main risk factors that aggravate the development of complications and poor outcomes. Pain relief by offering strong systemic analgesics is the cornerstone in cessation of TOA toxicity progression.

## Data Availability

No datasets were generated or analysed during the current study.

## Abbreviations

BCVA: Best-corrected visual acuity
TOA: Topical ophthalmic anesthesia
OTC: Over-the-counter
